# A Goal Scoring Probability Model for Shots Based on Synchronized Positional and Event Data in Football (Soccer)

**DOI:** 10.3389/fspor.2021.624475

**Published:** 2021-03-29

**Authors:** Gabriel Anzer, Pascal Bauer

**Affiliations:** ^1^Sportec Solutions AG, Subsidiary of the Deutsche Fußball Liga (DFL), Munich, Germany; ^2^Institute of Sports Science, University of Tübingen, Tübingen, Germany; ^3^DFB-Akademie, Deutscher Fußball-Bund e.V., Frankfurt am Main, Germany

**Keywords:** expected goals, XG, positional data, event data, applied machine learning, football, soccer, sports analytics

## Abstract

Due to the low scoring nature of football (soccer), shots are often used as a proxy to evaluate team and player performances. However, not all shots are created equally and their quality differs significantly depending on the situation. The aim of this study is to objectively quantify the quality of any given shot by introducing a so-called *expected goals (xG)* model. This model is validated statistically and with professional match analysts. The best performing model uses an extreme gradient boosting algorithm and is based on hand-crafted features from synchronized positional and event data of 105, 627 shots in the German Bundesliga. With a ranked probability score (RPS) of 0.197, it is more accurate than any previously published expected goals model. This approach allows us to assess team and player performances far more accurately than is possible with traditional metrics by focusing on process rather than results.

## 1. Introduction

In professional football (soccer), only 1% of all attacking plays and only around 10% of all shots taken end up in a goal (Pollard and Reep, 1997; Tenga et al., 2010; Lucey et al., 2014). However, goals alone decide the outcome of a game and are the most common metric to judge both a team's and individual player's performance. For example, both the best goal scorers^1^ and the players with the most assists^2^ receive a lot of attention from experts and the media. Nevertheless, judging performances solely based on this binary metric (*goal* or *no goal*) loses a lot of information and places results over process. For example, the performance from an outstanding creative player could be made void by strikers missing all their chances.

For this reason, in football as well as in other sports, it has become typical to consider more granular process-based metrics. In baseball, scouts and experts focused their attention on *homeruns* or *hits* for decades until more complex evaluation metrics changed the assessment procedure of hitters' performance significantly (James, 1985). Another famous example is basketball: By calculating scoring probabilities of different shot locations (Reich et al., 2006; Chang et al., 2014; Harmon et al., 2016; Jagacinski et al., 2019), the NBA's shooting behavior changed significantly^3^. The high scoring nature of basketball enables clubs to go even further and to apply individual shooting efficiency models (Beshai, 2014). Similar shot prediction models were also developed for ice hockey (Macdonald, 2012) as well as for return plays in tennis (Wei et al., 2016) and table tennis (Draschkowitz et al., 2015).

The fact that football is the lowest scoring game of the above-mentioned sports, makes it harder to develop such models, because of the scarcity of data. Consequently, the rareness and therefore importance of goals makes such a metric even more relevant when assessing teams and players. As another consequence of this low-scoring nature, the role of shots as a success proxy within several studies in football is fortified (Spearman et al., 2017). However, assessing shots just by being successful or not is a too rough abstraction that warps reality. An *expected goals model* (hereafter *xG model*) tries to estimate the probability of any given shot being converted to a goal based on various different factors describing the shot. These probabilities can then be added up per team and yield a “result-agnostic” description of the teams' performance. The xG metric is well-established in the football analytics community (see Davis and Robberechts, 2020)^4^^,^^5^^,^^6^^,^^7^. Although to the best of our knowledge, no peer-reviewed journal publication has introduced a positional data-driven xG model, valuable work has been done in “gray literature” like master theses (Hedar, 2020; Rowlinson, 2020) and conference proceedings (Lucey et al., 2014). Rathke (2017) analyzed in total around 18, 000 shots from one season of Bundesliga and Premier League based on manually acquired shot annotations. Differentiating between four different shooting types *(open play footed shot, header, freekick, or penalty shot)*, Ruiz et al. (2017) built a multi-layer perceptron to predict shot outcomes based on roughly 10, 000 shots. Using a similar approach, Fairchild et al. (2018) tried to predict the goal scoring probabilities of 1, 115 non-penalty shots from 99 Major League Soccer matches, again solely based on event data.

**Figure 1:**
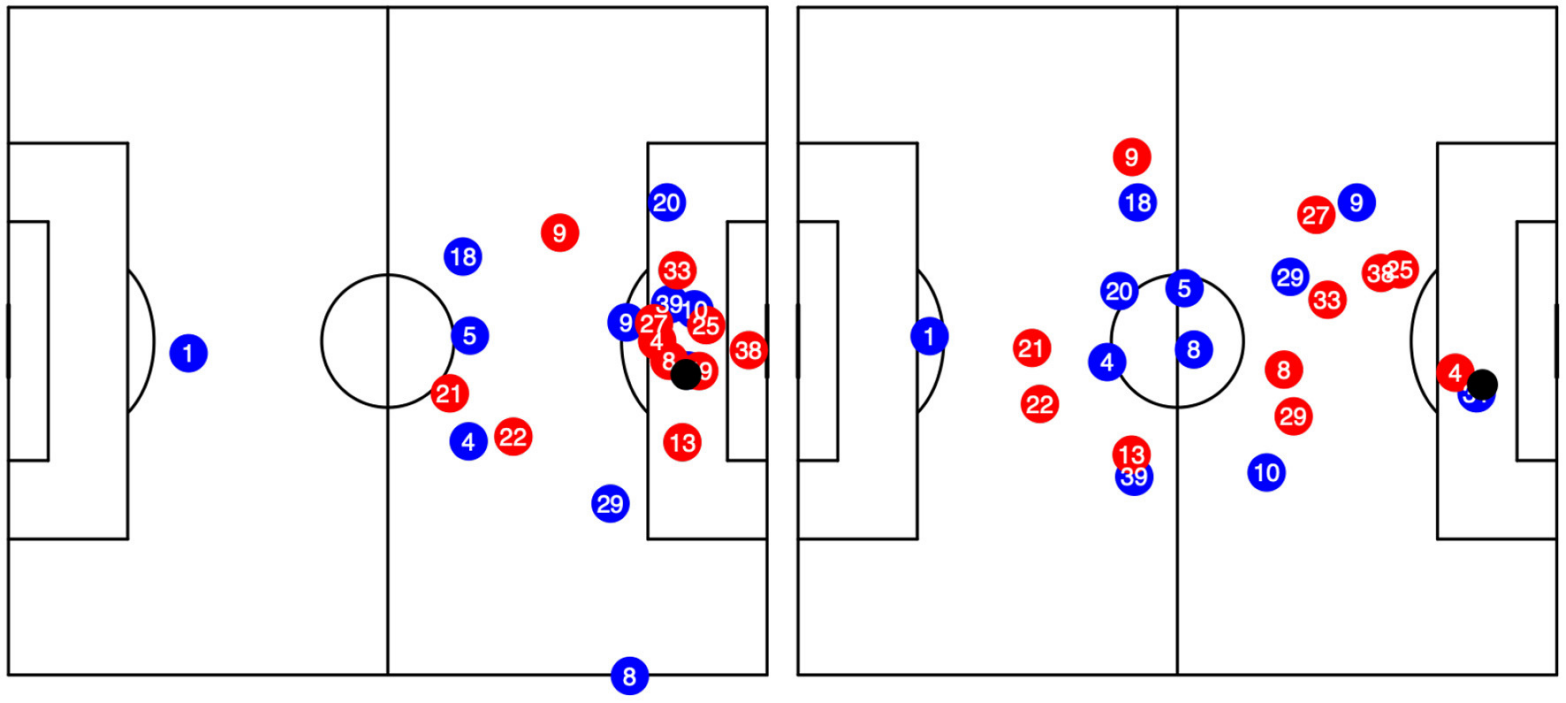
Player positions of two shots from roughly the same location, but different surrounding environments. In both cases, the blue team is playing from left to right.

Recent developments in technology allows us not only to make use of manually annotated event data *(shots, passes, goals with a manually assigned location)* but also accurate positions of all 22 players and the ball at up to 25 times a second. It is quite intuitive that the positioning of the defensive team, especially of the goalkeeper, has a crucial influence on the shot outcome (Lucey et al., 2014; Schulze et al., 2018). Figure 1 displays the positioning of relevant players during two shots occurring at similar spots. In the left figure, both a defender and the goalkeeper are in good position to block the shot, while in the right figure the attacker has already dribbled past the goalkeeper (#38) and defenders, and faces an easy tap-in into an empty goal^8^. However, this information is not covered in event data and thus not taken into consideration in the previously listed xG models. Lucey et al. (2014) were the first to estimate goal probabilities using event and positional data in their model. They used 10, 000 shots of the English Premier League.

In this paper, we will introduce a shot prediction model, utilizing event and positional data. The accuracy of this model is evaluated both statistically and based on the discussion with professional match analysts. We also incorporate their expertise both when defining the model's features and when interpreting their influence on the prediction. Additionally, we show how our model can support coaching staffs by introducing various use cases and applying them on one season worth of Bundesliga data.

The remainder of this paper is structured as follows. In section 2, we introduce the data and definitions. How event and tracking data are synchronized is described in section 3. Section 4 describes how the supervised prediction model is build, and finally, section 5 consists of two parts: practical applications (5.1) of our approach based on a season of German Bundesliga and a critical discussion of the results (5.2).

## 2. Data and Definitions

Like in most other professional football competitions, the German Bundesliga systematically collects positional and event data on a league-wide level in a pre-defined and thus consistent format. *Positional data*—often also referred to as tracking or movement data (Stein et al., 2015)—provides the positions of all players, referees, and the ball related to the pitch boundaries with a frequency of 25 Hz. These data are gathered by an optical tracking system, which captures high-resolution video footage from different camera perspectives. On the other hand, *event data* are manually acquired by trained operators live during the match. Among other things, this event data contain many details about basic events, such as passes, shots, fouls, saves, and so on including the involved players or special characteristics.

Since shots are an important statistic in football, the event data in the Bundesliga describe them with more than 20 attributes. For example, the collector differentiates between three basic shot types *(leg, header, other)* or six different scenarios how a player controlled the ball before taking a shot *(direct, volley, two touches, dribbling* > 10 *m, dribbling* < 10 *m, set-piece)*.

In this investigation, we make use of 105, 627 shots from German Bundesliga and 2^nd^ Bundesliga of the seasons 2013/2014 until 2019/2020. The event data were collected according to the official Bundesliga match-data catalog^9^, and the optical tracking data were provided by Chyronhego's TRACAB system^10^.

Due to a growing availability of optical tracking systems in football, several studies have been conducted to evaluate their accuracy (Redwood-Brown et al., 2012; Linke et al., 2018, 2020; Linke, 2019; Taberner et al., 2019). In Linke et al. (2020), the two versions of the TRACAB system (*Gen 4*/*Gen 5*)^11^ were compared to an accurate ground truth measurement^12^. Both systems achieved a diversion of < 10 cm from the ground truth system (RMSE Gen 4: 0.09 cm, Gen 5: 0.08 cm). A non-peer reviewed study confirmed these results^13^. All above-mentioned evaluation studies focused on player detection, whereas the detection of the ball—probably the hardest challenge for optical tracking systems—is not covered.

To the best of our knowledge, no scientific study evaluated the quality of event data. However, in the German Bundesliga the acquisition follows an elaborate quality assurance process. Critical information is double-checked manually live (e.g., goals and red cards). Finally, an independent person inspects and adds additional information (e.g., event locations) to all acquired event data after the match.

## 3. Making Use of Both Positional and Event Data

### 3.1. Synchronizing Shots With Tracking Data

A major challenge when attempting to use both tracking and event data is that they are generally not aligned. This is due to the fact that they come from different data providers and/or acquisition methods, one specialized in logging events manually according to catalog of set definitions (i.e., what is considered a shot or a tackling) and the other focusing on extracting player positions through, for example, computer vision algorithms. This leads to two potential issues when synchronizing the data:

- The manual collected event time stamps are prone to human errors, e.g., reaction time, distractions, and decision time, leading to time offsets of up to 20 s based on our investigations.
- The two systems use their own clock, causing systematic offsets between the two sources.

**Figure 2:**
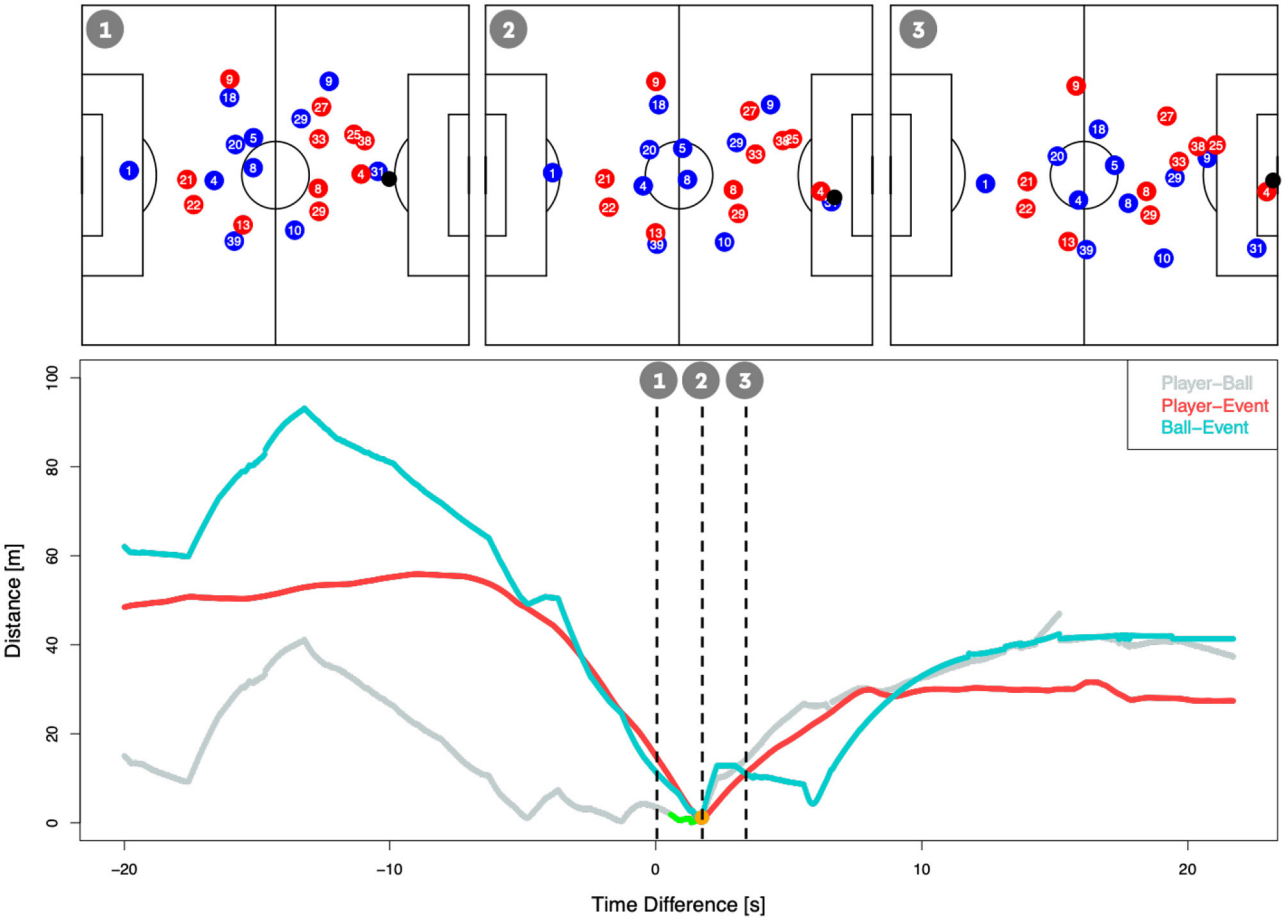
Relevant metrics for the synchronization over time. The green points highlight the time interval where we detect a potential individual ball possession sequence. The orange point indicates where the shot event was finally detected.

For these reasons, a “naive” synchronization—using the time stamp from the event data—to identify player positions at the time of an event leads to large inaccuracies. The upper plots in Figure 2 display the coordinates of the players and the ball at the different moments of the scenario from Figure 1 (right plot). The scene describes Kevin Volland's (Bayer Leverkusen) 1:0 against Borussia Dortmund (BVB) at the 14th matchday in the 2017/2018 season:^14^ The upper right plot in Figure 2 displays the shot time stamp tagged in the event data, which is roughly 2 s after the time stamp our synchronization suggests the shot took place (upper middle plot). The upper left plot in Figure 2 shows the positioning of the players 2 s prior to that. As one can see, the situations are drastically different ranging from a distant dribble to a player celebrating his goal. The figure underpins that a shift of a few seconds in the synchronization can have a massive impact on the features used for the xG calculation, like the shot location or the goalkeeper position.

**Figure 3:**
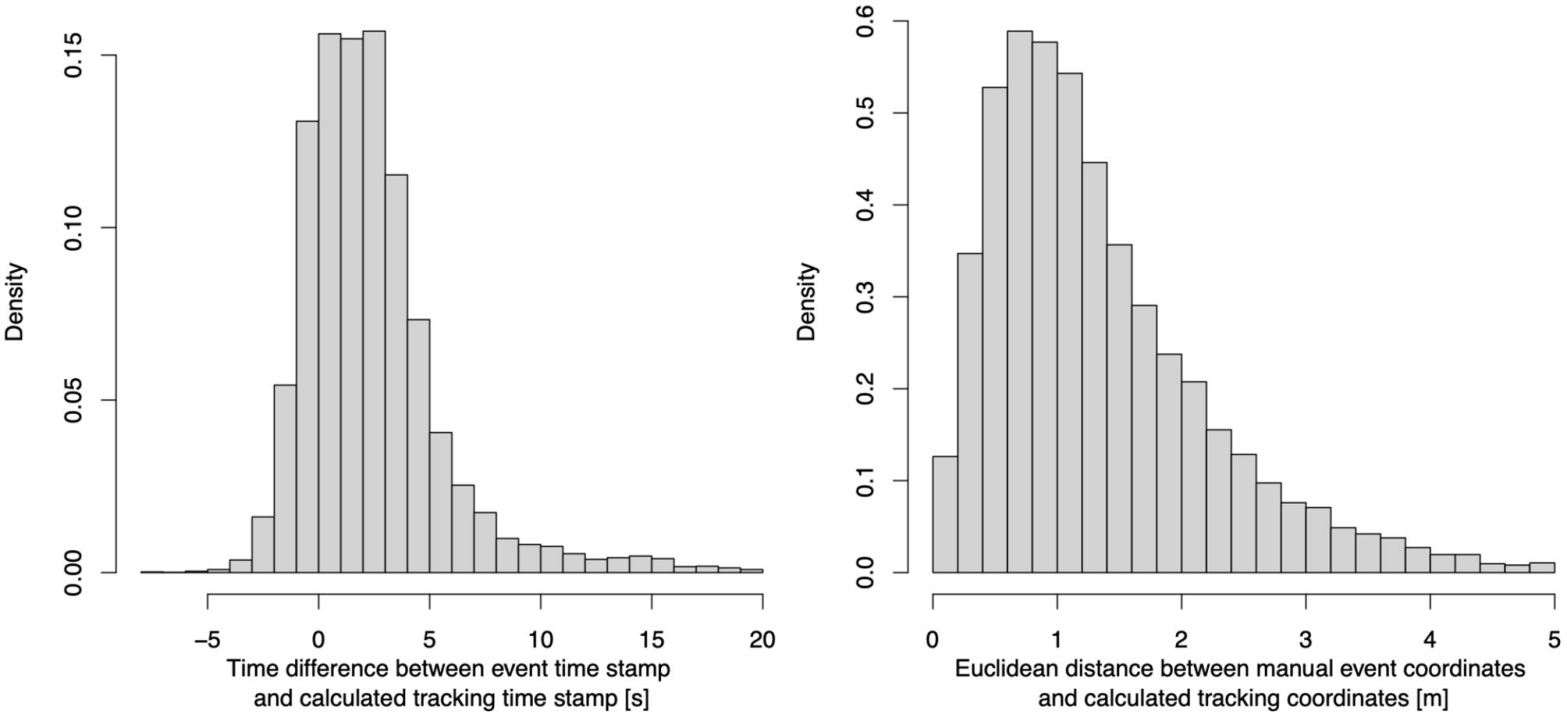
Time stamp **(left)** and shot location **(right)** differences between event and synchronized time stamps.

Therefore, we developed a synchronization algorithm tackling both issues. As a first step, we shift all tracking time stamps by the time difference between the kick-offs in both data sets. This resolves issue (b) and furthermore reduces a potential systematic delay in the manual event collection. In order to tackle issue (a), we compute several features that help to determine when a particular shot could have happened in the tracking data. First, we determine when the shooting player was in ball possession. We define potential individual ball possession sequences as the time interval when the player is in close proximity to the ball—our subject experts suggested 2 m as a cut-off, which is in line with Linke et al. (2018). Next, within each possession window, we identify the frame with the maximum acceleration of the Euclidean distance between player and ball. This aims to identify the exact moment where a shot occurred. Lastly, since there are potentially many situations that fulfill the above-mentioned criteria, we identify which best matches the event description. For that we compute Euclidean distances between the player and ball, the player and the manual collected event location as well as between the ball and the manual collected event location. Additionally, we compute the time difference between the (shifted) tracking time stamps and the manual collected event time stamp. We compute a weighted sum of these features, and the one frame out of the solution space that minimizes this weighted sum is chosen. The weights were obtained by performing a grid-based search that aimed to optimize accuracy of the synchronization on a manual labeled test set. The lower part of Figure 2 shows how these features behave in the 20 s before and after the exemplary shot described above. When we applied this synchronization algorithm on the full data set of six seasons, the event shot times had an average absolute offset of 2.3 s (≈57 frames) from the synchronized frame. Figure 3 displays histograms of the differences in timing (left) and locations (right) of each shot.

### 3.2. Evaluation of the Synchronization

In order to evaluate the accuracy of the synchronization, we manually annotated the timing of total 219 shots of the nine matches from matchday one of Bundesliga season 2018/2019. First, a full 90 min video animation of the 2D tracking data was created for each match. As a ground truth, we used a tactical video feed, which is filmed manually with an angle to capture all outfield player (and the most relevant goalkeeper). Additionally, for each match a xml-file^15^ containing all shot-events, and the kick-off was produced. Next, we used the kick-offs in all three data sources to synchronize them manually as accurately as possible using Hudl Sportscode^16^—a dedicated tool for football video analysis with functionalities to combine different video sources and data sources (i.e., event data can be imported via xml-files). For each shot, we stop the video at the exact moment the shot occurred—defined as the first frame when the ball left the shooter—and extract this time point using Sportscode functionalities.

We now use these labeled shot timestamps as the ground truth and compare them with both, the results from our synchronization, and the event timestamps. Our synchronization displays an average absolute offset of 0.23 (±0.49) s, while the event timestamps differ by 1.82 (±4.06) s. Out of the 219 shots, we were able to synchronize 218, and 210 (95.9%) of these shots were < 0.3 s apart from the ground truth^17^. In contrast, only 63 (28.8%) of the event timestamps were within 0.3 s of the ground truth. It is evident that generally this synchronization is far superior to event timestamps. Two exemplary situations for a successful and an unsuccessful shot synchronization can be found here^18^^,^^19^.

When a shot cannot be synchronized, it is typically due to either tracking data quality issues (e.g., the ball is poorly tracked, and never gets close to the player taking the shot, or two players were swapped in the tracking data) or event data quality issues (e.g., the wrong shooter is identified). To ensure that the quality of the input data is as high as possible, all shots that could not be synchronized at all were excluded from further analysis. Over the entire data set, this was the case in 3.4% of the shots.

All together, the synchronization of positional and event data presents a tremendous improvement for the analysis of shots, and could potentially be extended, using a similar algorithm, to other event types, like passes or tacklings. As we have seen above, misidentifying the shot time just slightly can cause a stark misrepresentation of its surrounding circumstances, and consequently affect the xG value significantly.

## 4. Expected Goals Modeling

### 4.1. Hand-Crafted Feature Extraction

To feed the supervised machine learning model, features influencing the goal scoring opportunity were defined together with professional match analysts from Bundesliga clubs and the German national team. A description of all features can be found in Table 1. In order to make full use of the synchronization of our two data sources, the features are based on both event and tracking data. The goalkeeper positioning is included in two features: We check whether they are in the line of shot, defined as the triangle between the shot location and the two posts, which is also the baseline for our shot angle calculation. Second, the distance between the goalkeeper and the goal is used as features in our model. The defending players' positions, either threatening to block the shot or applying pressure on the shooter, are also taken into consideration. Similarly to the goalkeeper feature, we count the number of defenders in the line of shot. Based on the logic from Andrienko et al. (2017), we calculate the total amount of pressure on the shooter aggregated over all defending players, as well as the maximum individual pressure on the shot-taking player. For both pressure metrics, we additionally compute the differences to the expected pressures given the shot location. Furthermore, the speed of the shooter, while taking the shot, is integrated in our model.

**Table 1:** Features derived from synchronized positional and event data used to train our model.

**Feature**	**Value**	**Description**
Shot location	Numeric	The x, y and the z-coordinate of the ball at the time of the shot are used for several features, such as angle and distance to goal center.
Speed of player taking the shot	Numeric	The speed of the player attempting the shot, at the time of the shot (in *km*/*h*).
Defenders in the line of the shot	Numeric	The number of defenders in the line of the shot.
Goalkeeper position	Numeric	The position of the goalkeeper is used for two different features, describing whether they are in the line of shot and their distance to the goal.
Pressure on the player taking the shot	Numeric	Various metrics describing the pressure that the player was under while attempting the shot, at the time of the shot Andrienko et al., 2017.
Type of shot	Categorical	Describing the body part used for the shot *(Head, leg* or *other)*.
Taker ball-control	Categorical	Describes how the player taking the shot gained control of the ball before/when taking the shot *(volley, controlShot, dribblingLess10m, dribblingMore10m, setPiece)*.
After freekick	Categorical	Indicates whether the shot followed a freekick.
Freekick	Categorical	Describes whether the shot is a direct freekick or not.

### 4.2. Predict the Scoring Probability as a Supervised Machine Learning Task

For a total of 105, 627 shots, all features from Table 1 were calculated based on the synchronized positional and event data. Since the features *shot type* and *freekick* significantly influence the contribution of all other features, we split our problem into three subtasks: the prediction of goal scoring probabilities of open play leg-shots, headers, and direct freekicks. Per subtask, the optimal set of features was explored. Consequently, for all three subtasks we trained several supervised machine learning models based on 81, 462 open play leg-shots, 18, 748 headers and 5, 417 direct freekicks, respectively, labeled by the information whether the shot ended up in a goal (1) or not (0). For each subtask, the shots were randomly split into 60% training, 20% validation, and 20% test data sets. To avoid over representing teams or scores, this split was conducted for every match separately. The final model, shown in Table 1 (row 5), describes the combination of our three submodels. To investigate the efficiency of the division into the three subgroups, another model is trained based on all 105, 627 shots taking all features from Table 2 including the information whether the shot was a header, a leg-shot from open play or a direct freekick.

**Table 2:** Statistical evaluation of the expected goal model outcome.

	**Model**	**Precision**	**Recall**	**AUC**	**RPS**
1	Gradient boosting (all situations)	0.646	0.181	0.822	0.196
2	Logistic regression	0.611	0.108	0.807	0.160
3	ADA boost	0.548	0.201	0.816	0.076
4	Random forest	0.611	0.163	0.794	0.165
5	Gradient boosting combined	0.665	0.164	0.823	0.197
	*Leg-shot model*	0.668	0.171	0.825	0.201
	*Header model*	0.655	0.161	0.813	0.187
	*Direct freekick model*	–	0	0.830	0.099
6	Chance evaluation model	0.516	0.420	0.688	0.170
7	Event data based model	0.587	0.098	0.772	0.118

Various standard supervised machine learning models were trained on the training data set, hyperparameters were optimized on the validation data set and the models' accuracy's were evaluated on the test data set. Naturally, the necessary hyperparameters depend on the machine learning algorithm. In the case of the extreme gradient boosting model (hereafter referred to as XGBoost), the parameters we optimized are as follows: *Learning rate*: controls the step size used per update; *Max depth*: limits the depth of the tree; *Subsample*: controls number samples applied to the tree; *Min child weight*: controls instance weight of a node. For the optimization, we applied Bayesian tree-structured Parzen Estimator hyperparameter optimization approaches for the gradient boosting model (Bergstra et al., 2011; Dewnacker et al., 2016; Wang, 2019).

For several models in Table 2, we calculated SHAP values per feature (Roth and Thomson, 1988; Lundberg and Lee, 2017; Rodríguez-Pérez and Bajorath, 2020). In several applications, using SHAP values^20^ instead of standard gain values has proven to be beneficial (Antipov and Pokryshevskaya, 2020; Ibrahim et al., 2020; Meng et al., 2020).

In order to get a better understanding of the resulting model's accuracy, we implemented two simple models as a baseline models. The first one uses an attribute that is collected for every shot (*chance quality*). This manually collected attribute can contain one of the following two values: sitter or chance. The very simple model now assigns each shot the average conversion rate of the corresponding class. So all shots labeled as *chances* are assigned a value of 0.063, while the remaining shots labeled as *sitters* receive a value of 0.548. The second baseline model uses all the event data based features from Table 1 (namely *Shot location, Type of shot, Taker ball-control, After freekick*, and *Freekick*), and train a XGBoost model using these features.

### 4.3. Statistical Evaluation of the Shot Prediction Model

The first two validation metrics (precision and recall) presented in Table 2 evaluate the outcome of a classification problem. A goal classified with an xG above 50% is classified as a true positive, whereas an unsuccessful shot with an xG below that threshold is defined as a true negative. Thereafter, a recall of 1 could simply be achieved by assigning each shot an xG value above 50%. To incorporate both the true positive and the false positive rate depending on the threshold into our evaluation, we also use the area under the receiving operator curve (*AUC*) as an error function (Daskivich et al., 2018). However, it is our objective to assess the accuracy of the underlying goal scoring probabilities and not just of a binary classification (goal or no goal). While this is possible with the AUC, using the ranked probability score (*RPS*), as presented in Murphy (1970), fulfills this purpose better, especially for imbalanced data sets.

By splitting up the shots into two groups (chances and sitters), the chance evaluation model (Table 2, row 6) achieves a good balance between precision and recall. While this relatively simple model already achieves a somewhat satisfactory RPS of 0.170, the human-made classifications are possibly biased by the shot outcomes. This label is therefore not used as a feature for the remaining prediction models. For the event data based model, the extremely low recall can be interpreted as follows: The model predicts xG value below 50% for most of the shots that actually end up as goals. However, the AUC shows that the event-based model yields more granular predictions than the chance evaluation model. In the direct freekick submodel, no xG prediction exceeds 50%, and therefore its precision is undefined.

Shots are non-deterministic, at the time of the shot, meaning that no model can have a 100% accuracy predicting whether any given shot will score. But what we can expect from our model predictions is that they converge over a large sample. To verify this, we looked at the first 54 matches (matchday one through three) of the 2020/2021 season in Bundesliga and 2nd Bundesliga. Out of the 1, 357 shots, 150 found the back of the net and our model predicted an aggregated xG value of 151.6.

**Figure 4:**
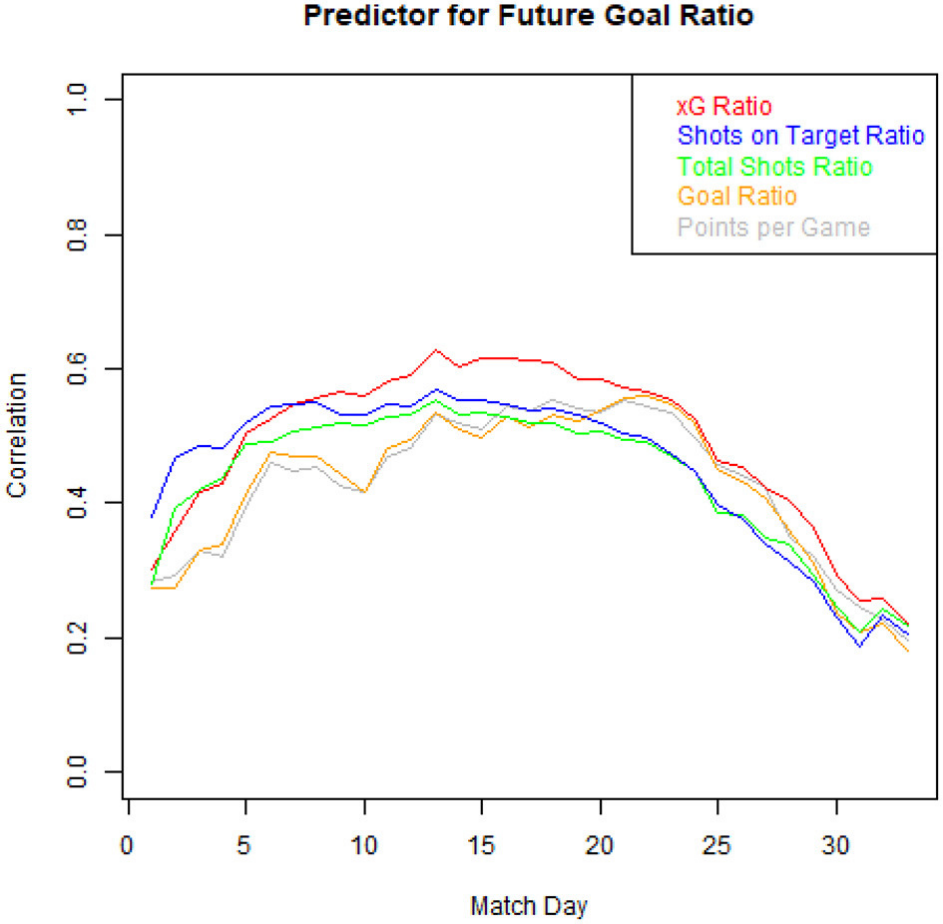
Correlation between a team's future goal ratio after a certain matchday and an aggregated metric before said matchday (average of all seasons 2013/2014–2019/2020).

Estimating a team's true strength or its future performances is a crucial unsolved problem in football with many potential use cases (Goes et al., 2019). Both shots on target, two well-established metrics in the literature, have been used for this context (Lamas et al., 2014). Figure 4 displays in which scenarios our xG values fulfills this task better than traditional approaches. It looks at how well you can predict a team's future rest of the season goal ratio (defined as the difference between goals scored and goals conceded) after a certain matchday, by only taking into account one aggregated metric before said matchday. On the *y*-axis, the correlation between the future goal ratio and the respective metrics (see legend) before that matchday (*x*-axis) is shown. Consistently, over all considered seasons a team's historic xG values are able to predict future results better than traditional metrics, especially between matchday 10 and 20. Additionally, we found that in 73.3% of all matches (excluding draws), the winner had a higher xG value^21^, while only in 56.2% of these games, the winning team had more shots, than its opponent.

**Figure 5:**
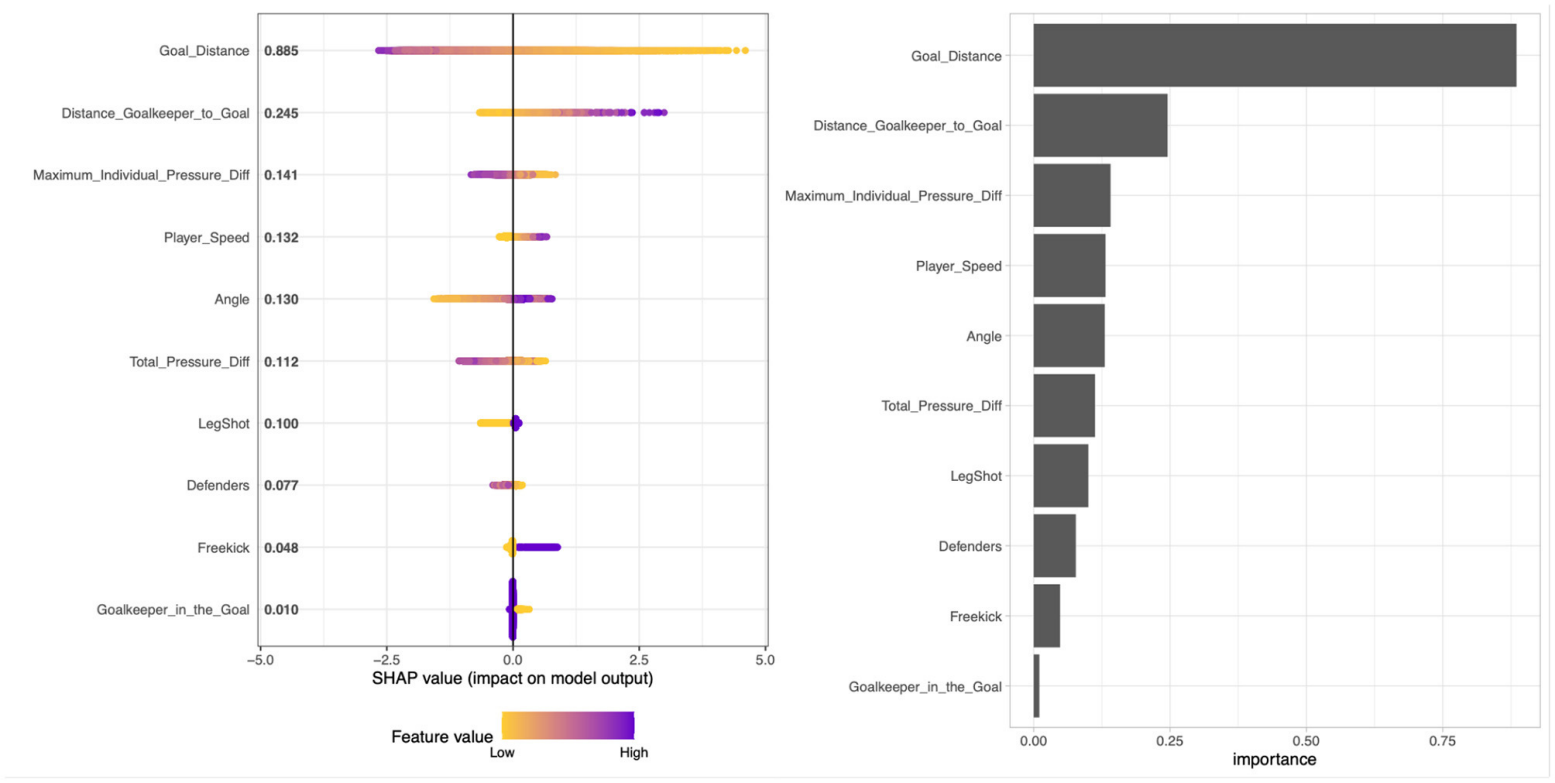
Feature importance according to Shapley values displayed as a SHAP summary plot **(left)** and global feature contributions by the mean SHAP value across all samples **(right)**.

**Figure 6:**
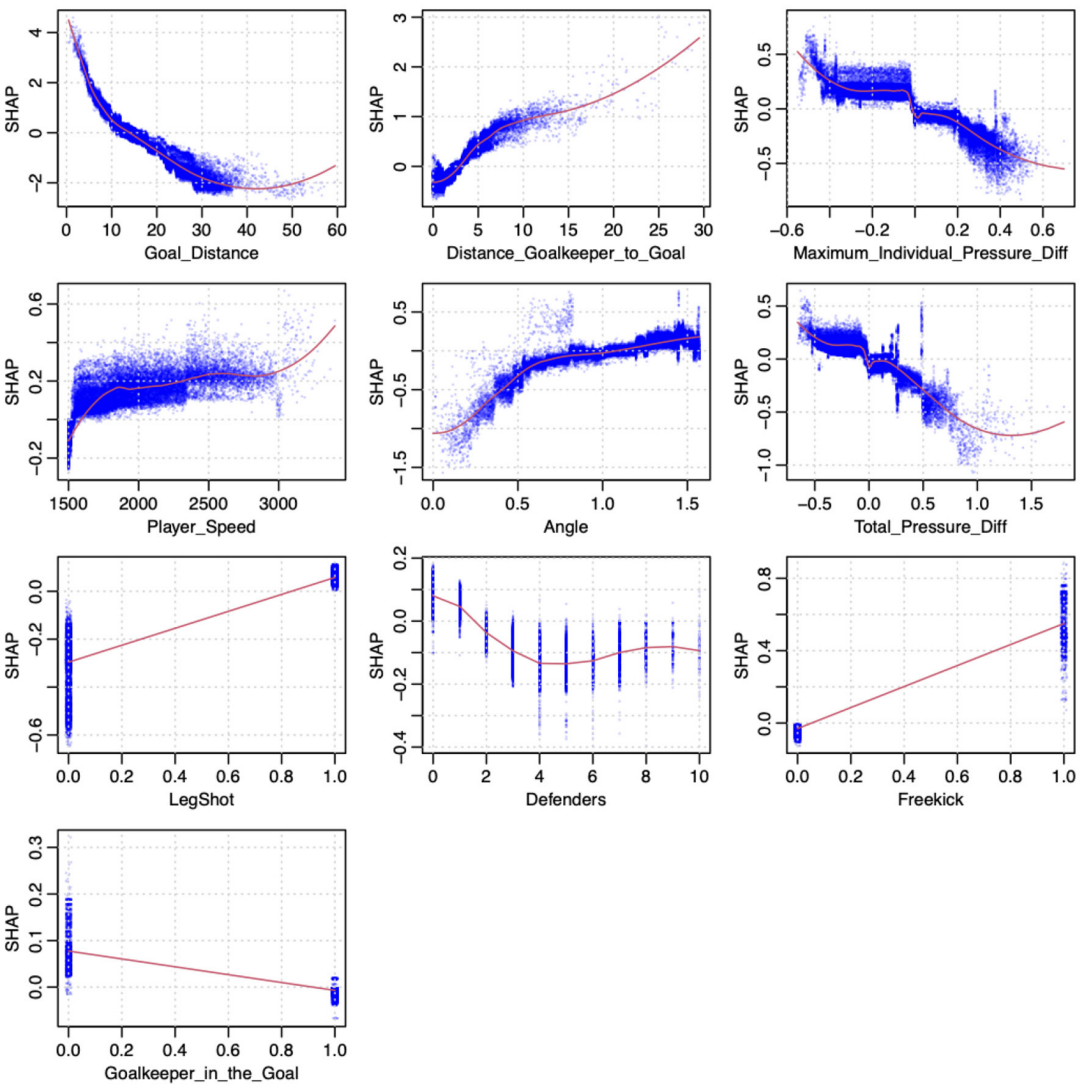
SHAP dependence Plot. For each shot, the respective feature value is plotted on the x-axis vs. the corresponding Shapley values on the y-axis (distance is displayed in meter, and speed is shown in meter per hour).

Next, we analyze the features' influence on the predicted goal scoring probability. In the following, we discuss the overall feature importance of our gradient-boosting model trained on all shots with the subcategories as features (Table 2, row 1). Figure 5 displays the overall influence according the respective SHAP values per feature on the right, which can be interpreted as an aggregated quantification of the feature's influence. The SHAP values show that the most crucial factors are the shot location *(Goal Distance, Angle)* and the goalkeeper position *(Distance Goalkeeper to Goal)*. *Maximum Individual Pressure Diff*, defined as the difference between the actual pressure and the average pressure given the shot location, has the third highest influence on the predicted values. In Figure 5 (left plot), the *x*-value of each colored dot displays how a feature influences the model, whereas the color scaling describes the value of the respective feature. Both a flat line and a smooth change of colors (from left to right or vice versa) indicates a roughly linear correlation between the feature value and the model outcome. In Figure 6, this relationship between the feature values (*x*-axis) and influence on the model (*y*-axis) is shown more granularly. Although the red line shows a regression, the dispersion of the blue dots provide a deeper insight. Both the left plot in Figure 5 (smooth decrease of the colored dots from left to right) and Figure 6 (red line) shows that the goal distance has an almost linear impact on the predicted values. However, if the distance to the goal is very high, influence relies more on other features, as can be seen by the growing dispersion of the blue dots. The importance of the number defenders in the line of the shot (here *Defenders*) underpins the relevance of using positional data, including all opposing players' positions. Looking deeper into the SHAP distributions of this feature, Figure 6 shows an almost linear decrease of the average SHAP value over all shots from zero to four defenders in the line of shot. For more defenders in the line of shot, the average SHAP value—describing a proxy for the features influence—remains mostly constant. In Figure 6, the feature *Goalkeeper in the goal* underpins our practitioners' intuitive assumption and can be interpreted as follows: If the goalkeeper is not in the line of shot, it increases the xG value significantly.

Again, most of this information would not be available in event data, which highlights the benefit of using both event and positional data once more.

### 4.4. Evaluation by Subject Matter Expertise

In several workshops with match analysts from Bundesliga clubs and the German national team, the features were defined and ranked according to the estimated influence. These estimations were compared with the above calculated feature importance. Additionally, the SHAP value dispersions and interpretations were discussed in detail. Besides a lot of agreement from practitioners, some statistical results—, e.g., the influence of 4–10 players in the line of shot—were discussed intensively among experts. To evaluate the plausibility of our model from a practitioners perspective, a workshop with selected (assistant) coaches of Bundesliga and 2nd Bundesliga clubs was conducted. For the recently concluded season, the coaches were asked to classify their matches into four categories: *deserved* or *undeserved* victories, draws, or losses as in **Figure 8**. Afterwards, we compared their labels to the ones produced from our xG model. With a category-accordance of more than 85% (in total 102 matches with 293 goals), practitioners characterized our approach as a helpful tool to assess individual shot qualities and the overall performance of a team.

## 5. Application and Discussion

### 5.1. Applications

For the following section, we consider the 2019/2020 season of the German Bundesliga, with in total 306 matches, 954 goals, and 5, 450 shots. We describe how the goal scoring probability *xG*(*S*) model for a given shot *S* is aggregated over a season to evaluate teams and players further:

$$\begin{array}{l} xG_{\text{agg}}\left(\text{Team}/\text{Player}\right)=\underset{S_{i}\in \text{Shots}}{\sum }xG\left(S_{i}\right) \end{array}$$

Own goals are not a subtype of a shot event, but rather a separate event type with different attributes. Therefore, they are excluded from our xG calculation. Penalties are assigned an xG value of 0.766, which is the average conversion rate in the Bundesliga history. In the case of so-called double-chance, situations in which a first shot is blocked, but is immediately followed up by a rebound shot, we calculate xG values for each shot. But when we aggregate the team level xG values, we do not want to simply add them up, because it could lead to situations where a teams xG value for small time-window could exceed 1. Therefore, given a double-chance *S*, defined as two shots within 5 s, we compute the overall probability as:

$$\begin{array}{l} xG\left(S\right)=xG\left(S_{1}\right)+\left(xG\left(S_{2}\right)*xG\left(\bar{S_{1}}\right)\right) \end{array}$$

#### 5.1.1. Teams

**Figure 7:**
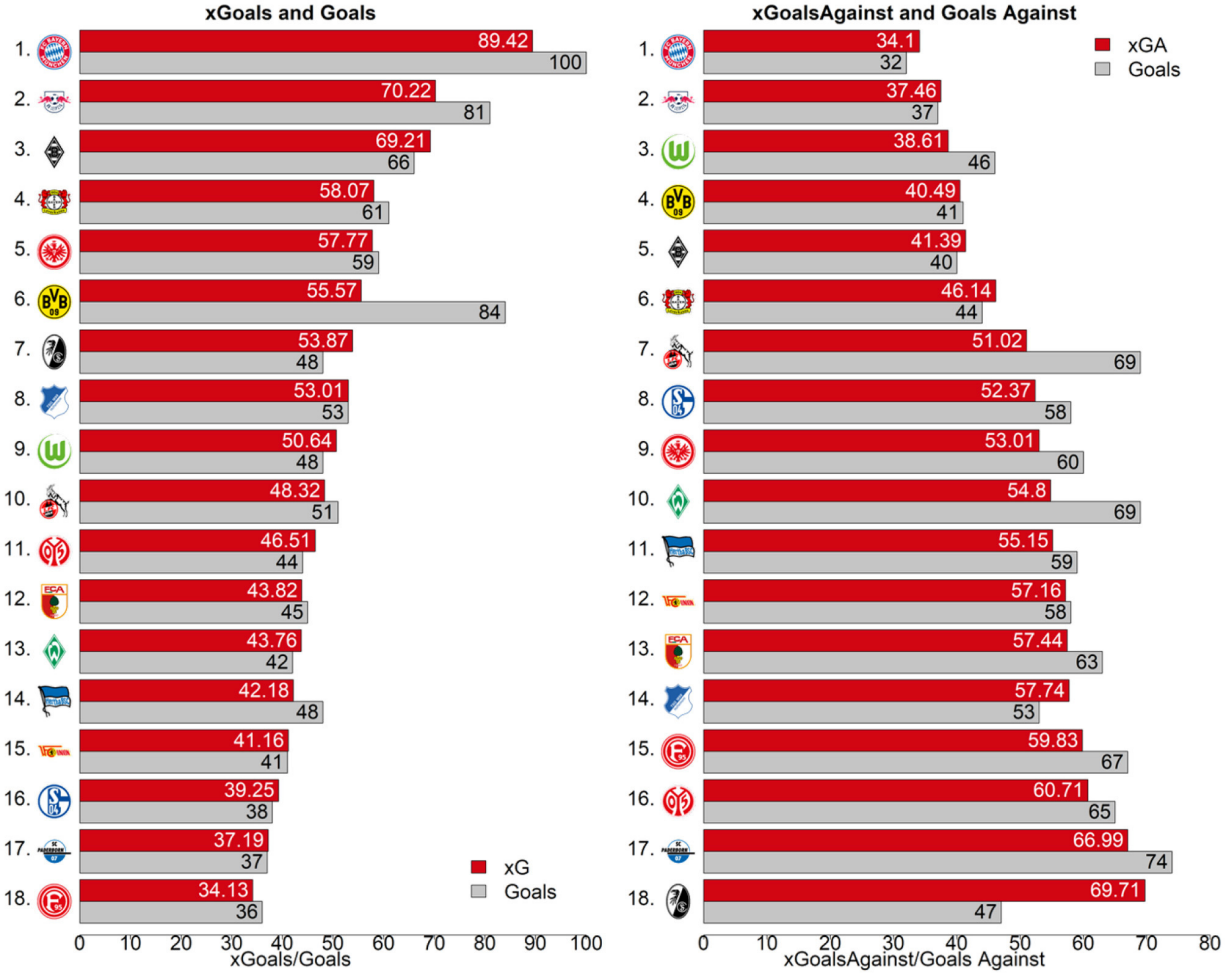
Bundesliga 2019/2020 season ranking with aggregated xG and the actual number of goals (xG red, actual goals gray).

**Figure 8:**
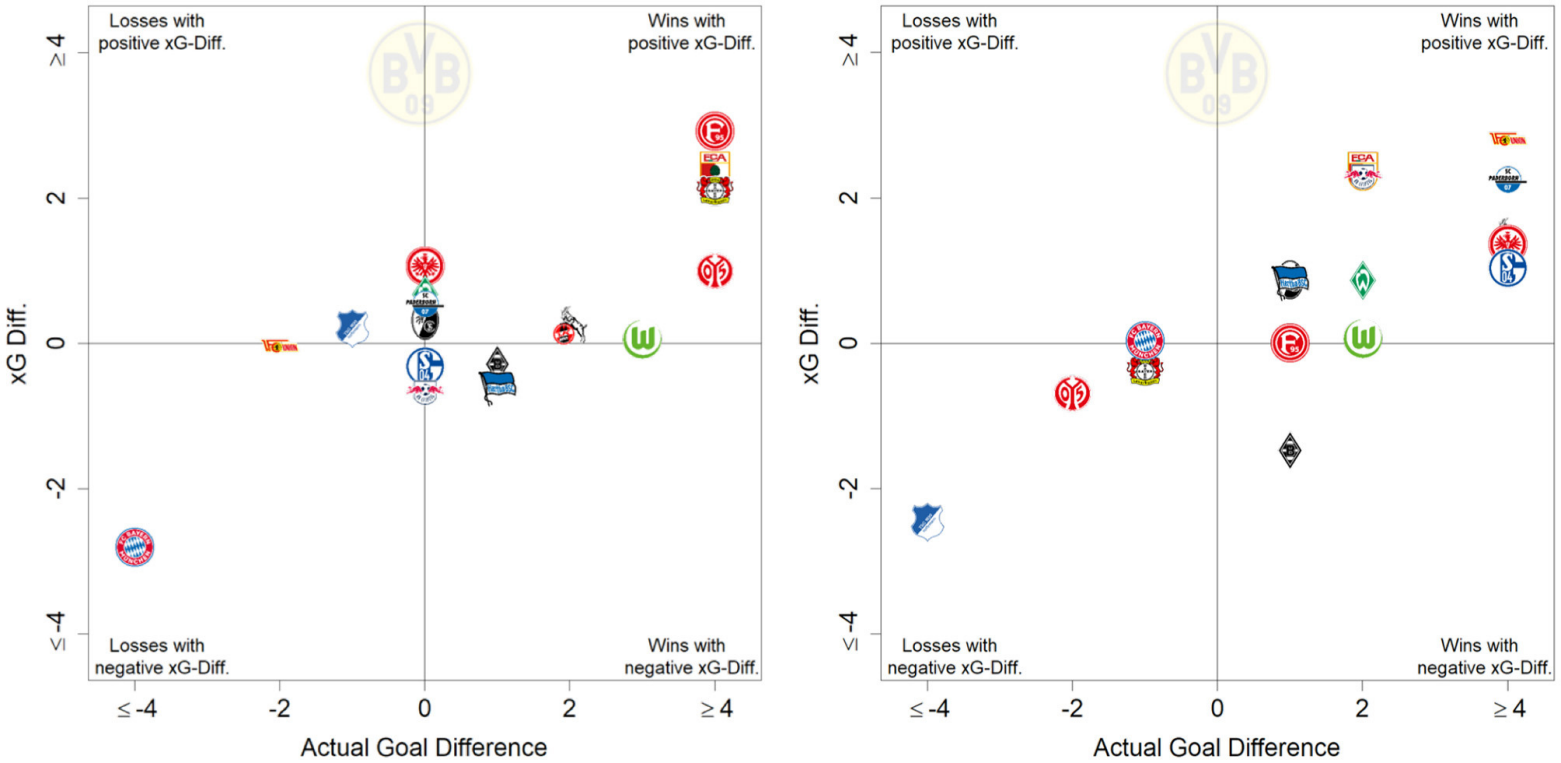
Season report of BVB in season 2019/2020 showing efficiency of BVB matches according to the underlying xG values.

Figure 7 displays how many goals each team scored and conceded in comparison to the aggregated xG values our model computed. Consequently, for the 2019/2020 season, BVB (sixth place in the left ranking of Figure 7) scored roughly 30 more goals than the sum of all the respective shots' xG values would suggest. Figure 8 provides a closer look at BVB efficiency on a match level. Comparing actual goal differences to the xG differences, the upper right quadrant could be interpreted as *deserved* wins, where BVB created more promising shot opportunities than their opponents. Matches on the lower right could be interpreted as lucky wins, e.g., the return match^22^ against Borussia Mönchengladbach (black and white hatched diamond logo in the bottom right of the left figure).

**Figure 9:**
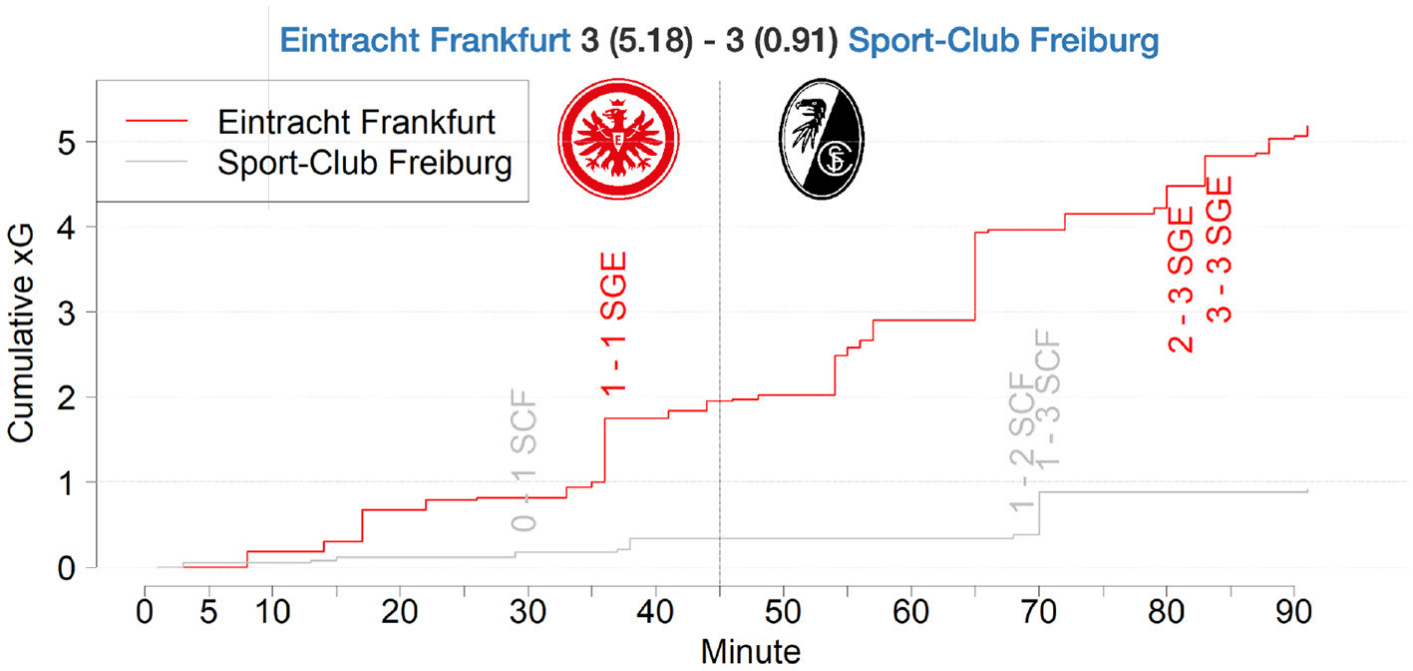
xG match report of a Bundesliga match between SC Freiburg and Eintracht Frankfurt in season 2019/2020.

Another match, where our model would have predicted a different result is displayed in Figure 9^23^. The graph shows the aggregated xG values per team over the course of a match. Although SC Freiburg displayed an extraordinary shooting efficiency, by scoring three goals out of three difficult situations, Eintracht Frankfurt created several high quality chances but only converted three of them.

**Figure 10:**
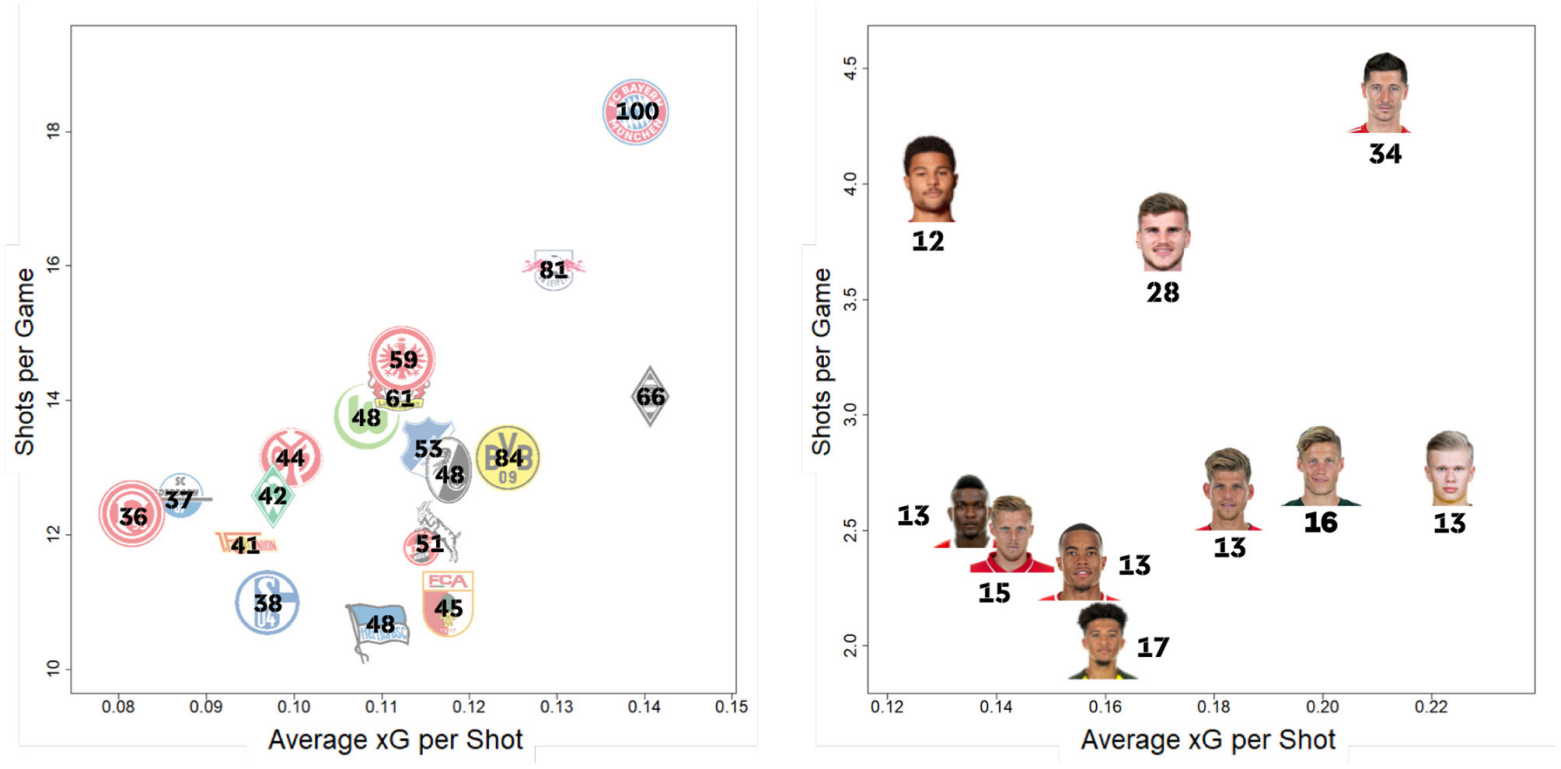
Quality vs. quantity of shots taken per team (left) and player (right). The total number of goals scored over the whole season per team and player is displayed in black.

Furthermore, our model can help match analysts examine a teams' shooting behavior. Figure 10 presents the number of shots taken vs. the average xG-value per team (left) and for the most scoring strikers (right). Although Fortuna Düsseldorf (red/white logo furthest left in Figure 10) had an average xG value (∅(xG)) of 0.08 in the 2019/2020 season, Borussia Mnchengladbach seems to take their shots only in cases of a clear scoring opportunity (∅(xG) = 0.14). FC Bayern Munich (red/blue/white logo top right in Figure 10), takes by far the most shots per game. However, with around four less shots per match, Borussia Mönchengladbach has a higher quality of attempts according to our xG model. Comparing FC Augsburg (red/white/green logo with *FCA* inscription) to Werder Bremen (green diamond logo with a white *W* as an inscription) shows two distinct patterns. While both teams had a similar number of aggregated xGs over the whole season (see Figure 7), Bremen tends to take more shots in less promising situations, while FC Augsburg emphasizes more on taking their shots in situations with a higher goal scoring probability. Having this information for the next opponent prior to each match can help teams to adapt their defending strategy depending on the opponent's shooting preferences.

#### 5.1.2. Players

Additionally, we can use player aggregated xG values, both for individual player performance analysis as well as scouting. Comparing Jadon Sancho to Serge Gnabry shows that both players—playing in similar positions and both with very successful teams—have strongly differing shooting patterns. Although Serge Gnabry (top left in Figure 10) takes the second most shots per match, Jadon Sancho (lowest in Figure 10) takes the fewest shots out of the top 10 scorers, but often in more promising situations according to the xG-values. Besides an overview of strikers shooting behavior in Figure 10, xG provides a lot more applications to quantify a player's offensive contribution more granularly than traditional metrics.

Since our xG model can be seen as an average across all Bundesliga players' shot efficiency, it can also be used to find players that convert shots at an above average rate. Using this approach, we see that Robert Lewandowski (upper right in Figure 10) outscored his aggregated xG value (29.6) by about four goals, scoring a total of 34 in the season out of his 140 shots (Table 3, row 12). While this is already an impressive feat, there were in total 11 players, outscoring their xG totals by a larger margin. Jadon Sancho (17 goals/53 shots/8.49 *xG*_agg_) and Erling Haaland (13 goals/34 shots/7.59 *xG*_agg_) lead this category and showed an extraordinary scoring efficiency.

**Table 3:** Players with the highest scoring efficiency in the German Bundesliga 2019/2020 season.

	**Name**	**Club**	**Minutes**	**xG**	**Goals**	**Shots**	**CR**
1	J. Sancho	Dortmund	2,386	8.49	17	53	2.002
2	E. Haaland	Dortmund	1,117	7.59	13	34	1.712
3	J. Cordoba	Köln	2,107	8.41	13	60	1.545
4	R. Hennings	Düsseldorf	2,598	9.92	15	71	1.512
5	A. Kramaric	Hoffenheim	1,496	8.61	12	42	1.393
6	T. Werner	Leipzig	2,934	20.79	28	122	1.346
7	R. Quaison	Mainz	2,727	10.72	13	69	1.212
8	A. Silva	Frankfurt	1,671	9.91	12	55	1.210
9	K. Havertz	Leverkusen	2,570	10.13	12	56	1.184
10	M. Reus	Dortmund	1,568	9.31	11	47	1.181
11	N. Petersen	Freiburg	2,588	9.44	11	54	1.165
12	R. Lewandowski	Bayern	2,888	29.57	34	140	1.149
13	S. Andersson	Union Berlin	2,821	11.68	12	64	1.027
14	S. Gnabry	Bayern	2,288	12.74	12	100	0.941
15	W. Weghorst	Wolfsburg	2,898	17.59	16	88	0.909
16	F. Niederlechner	Augsburg	2,858	14.93	13	82	0.870

*CR describes the conversion rate from xG to goals*.

### 5.2. Discussion

We present an xG model that performs better than any of the approaches discussed in the introduction. Rathke (2017) split the pitch into eight zones and trained a logistic regression on each, indirectly taking shot location and angle into consideration. However, their analysis was neither tested on unseen data nor took the positions of defenders and goalkeepers into consideration. By contrast, Lucey et al. (2014) did not only make use of positional data, but also displayed the improvements of the model accuracy. They split all shots into six different game-context situations *(open play, counterattack, corner, penalties, freekicks, set pieces)* and also learned a regressor for each. Their average error across all shots and scenes is 0.1439. In our final combined model (Table 2, row 5), this average error is 0.0928. As a combination of the larger data set (more than 100, 000 shots), our novel synchronization approach (see section 3) and the expert crafted features (see section 4.1) are possible reasons for this improvement.

However, xG models in football are not without flaws. An often criticized point is that they are not evaluating dangerous situations where no shot took place. While this criticism certainly has merits, most offensive actions end up in shots. The official Bundesliga event data include an event type *chance without a resulting shot*, describing situations, where a team was in a scoring position, but failed to attempt a shot. In our data set, this event occurs on average only 0.93 times per match, underlining that the impact non-shot opportunities have for measuring team performance is rather small. Additionally, as seen in section 5.1, evaluating team strength is not the only application of xG. Shot conversion on team/player level, average shot quality or even on a goalkeeper analysis are insightful use cases that only depend on actual shots taken. Nevertheless, several studies aim to tackle this problem, of noteworthy goal-scoring opportunities without shots, by computing so-called expected possession values (Link et al., 2016; Spearman, 2018; Fernández et al., 2019), but even these concepts are often build upon a well-calibrated xG model.

Following the logic of expected possession values, it is definitely a potential next step to break the contribution to a goal scored further down to the participating players and their actions. For instance, in the situation described in Figure 2 by assuming shots at several time-points, a simple rule-based approach using our xG model can quantify how much xG Volland added through his dribbling. Another popular extension of xG are expected assists (xA), which measure the likelihood that a pass leading to a shot becomes an assist, by assigning it the resulting xG value. This allows to quantify a player's shot assisting qualities independent of the final shooter's ability to score.

Both the synchronization and the inputs for the xG model heavily rely on the quality of the underlying data. Even for purely event data based xG models, Robberechts (2019) showed that their usefulness strongly depends on the event data quality. One of the parameters causing the biggest inaccuracy in the current model is the ball height. Small objects—like a ball—are hard to track based on video footage, especially due to confusion with replacement balls or other small white objects occurring in the stadium. For header shots, little differences in the ball height have a large impact on the ability of a player to control the placement of a shot causing inaccuracies for our current header model (Table 2, row 7). With a steady increase of video camera resolutions and object detection algorithms, we expect a significant improvement for ball tracking. This increase in data quality would likely improve shot synchronization results even further (see section 3.2) and consequently result in even more accurate xG models. Nevertheless, both for tracking data (including ball tracking) and for event data additional evaluation studies to ensure a high data quality for similar projects is essential. Although latest positional and event data provide accurate and detailed information about players, their body orientation and limb tracking could further improve the model's accuracy. For the header model in particular, heights and jumping altitude capacities could be taken into consideration as well.

The harmonization of tracking and event data is not a problem unique to football, which has been barely explored in the literature. In basketball, for instance, the two data sources^24^ are mainly used independently of one another (Tian et al., 2020), but as Manisera et al. (2019) noted the combination of both data sources is a crucial future issue. While our algorithm is optimized for football events, it could be adapted and applied to several other sports where both data sources are available.

An accurate expected goals model provides tremendous decision-making support for clubs: Creating many high-quality shooting situations is a crucial indicator of a good performance. To which extent these situations actually end up in goals often depend on random factors or luck. Consequently, a single final match result may not represent the actual team performance accurately. By quantifying a team's conversion rate (goals vs. xG) separately from their aggregated offensive contribution (created xG), clubs can evaluate the performance of their players, teams, and coaches objectively. Future research could even go one step further and explore how this work could affect the way the game is played. One could use our goal probabilities to determine numerically in which situations it is beneficial to shoot, and when one is better of risking an additional dribble or pass. Another area where the use of xG could be explored further are media applications: Recently, media and broadcasting have included xG values in their match coverage. For each goal occurring in German Bundesliga, different broadcasters have chosen to display our xG value seconds after the goal occurred^25^.

Now that the amount of data-driven approaches to support tactical analysis in football is increasing (Goes et al., 2020), more qualitative studies might help to underpin the statistical evaluation of models like xG. Although we present a first attempt toward an expert-based evaluation of our approaches (see sections 3.2 and 4.4), there is a lot of potential for further investigations, which could also serve to establish data-driven methods in the sport science and football community.

## 6. Conclusion

We present a meaningful proxy for goals scored in football, which helps to evaluate players' and teams' performance more accurately and objectively. Our xG model is based on a huge data set of cutting-edge and consistently acquired positional and event data that we combined using our own synchronization algorithm.

It exceeds traditional metrics significantly when evaluating strikers' (Table 3) and teams' (Figure 7) scoring efficiency, when evaluating single match performances (i.e., teams with higher xG win 73.3% of all not-drawn matches) and even when predicting future match results (Figure 3). It also allows us to evaluate assist performances of players independent of the striker's final touch. Additionally, several future potentials are shown for sport and data science research.

## Data Availability Statement

The data analyzed in this study is subject to the following licenses/restrictions: data are property of DFL/DFB e.V. and thus can not be shared publicly. Requests to access these datasets should be directed to Sportec Solutions AG, DFL e.V., DFB e.V.

## Ethics Statement

Written informed consent was obtained from the individual(s) for the publication of any potentially identifiable images or data included in this article.

## Author Contributions

GA was responsible for the implementation of the approach and involved in all discussions with practitioners. PB focused on the practical evaluation and the communication with practitioners. Both authors conducted the scientific studies together and were equally involved in the writing process of the manuscript.

## Conflict of Interest

GA was employed by the company Sportec Solutions AG and PB was employed by the company DFB-Akademie (Deutscher Fußball-Bund e.V).

## Footnotes

^1^https://www.goal.com/en-us/lists/cristiano-ronaldo-lionel-messi-pele-who-are-the-top-goal-scorers-/ynctx2o9fa371vi1x0dsgr0np (accessed July 10, 2020).

^2^https://www.givemesport.com/1534019-the-top-10-players-with-the-most-assists-in-europes-top-five-leagues-this-decade (accessed July 8, 2020).

^3^https://fivethirtyeight.com/features/how-mapping-shots-in-the-nba-changed-it-forever/ (accessed July 10, 2020).

^4^https://www.americansocceranalysis.com/home/2017/3/6/validating-the-asa-xgoals-model (accessed October 24, 2020).

^5^http://www.northyardanalytics.com/blog/2015/08/22/pitfalls-of-measuring-shooting-and-saving-skill/ (accessed October 24, 2020).

^6^https://www.optasports.com/services/analytics/advanced-metrics/ (accessed October 24, 2020).

^7^https://differentgame.wordpress.com/2014/05/19/a-shooting-model-an-expglanation-and-application/ (accessed October 24, 2020).

^8^The situation in the right plot is also displayed in Figure 2. The respective video can be found here: https://www.youtube.com/watch?v=UdvrKfsJISY&feature=onebox&t=1m08s (accessed October 24, 2020).

^9^https://s.bundesliga.com/assets/doc/10000/2189_original.pdf (accessed September 10, 2020).

^10^https://chyronhego.com/wp-content/uploads/2019/01/TRACAB-PI-sheet.pdf (accessed September 10, 2020).

^11^Note that the *Gen 5* system has been in use since season 2019/2020, while all prior Bundesliga seasons were tracked using the *Gen 4* TRACAB version.

^12^The ground truth was measured by a VICON system, using an optoelectronic motion capture system based on markers placed on the tracked objects. Further details about this system can be found here: https://www.vicon.com/. An evaluation study of that system can be found in Merriaux et al. (2017).

^13^The study was conducted by the *Fédération Internationale de Football Association (FIFA)* in close cooperation with the Victoria University (Melbourne, Australia). An overview of the study can be found here: https://football-technology.fifa.com/en/media-tiles/fifa-quality-performance-reports-for-epts/, the report of the *Gen 5* system can be found here: https://football-technology.fifa.com/media/172171/chyronhegoopt-fifa-epts-report-nov2018.pdf (accessed December 26, 2020).

^14^https://www.youtube.com/watch?v=UdvrKfsJISY&feature=onebox&t=1m08s (accessed September 10, 2020).

^15^Xml stands for *e****X****tensible* ***M****arkup* ***L****anguage* and is an established format to transfer complex data files.

^16^https://www.hudl.com/products/sportscode (accessed June 20, 2020).

^17^We use a range here, because both, harmonizing the different video and data sources and the manual selection of the shot timestamp, may cause slight time discrepancies.

^18^In the first sequence, actual match-footage of a scene is shown. The second shows a 2D animation of the same scene, with a frame-counter on top. This frame counter counts down till 0 where the shot happened and increases afterwards again. The third sequence combines both video sources together (see Supplementary Video 1).

^19^See Supplementary Video 2.

^20^The abbreviation **SHAP** stands for **SH**apley **A**dditive ex**P**lanation.

^21^On a match and team level the overall xG balance between the two teams is considered here. For both teams, we sum up the xG values per team of all their shots.

^22^https://www.youtube.com/watch?v=RUaORAinaoc&feature=onebox (accessed October 2, 2020).

^23^https://www.youtube.com/watch?v=jl1C0KsIqaQ (accessed October 2, 2020).

^24^In basketball, event level data are often referred to as *play-by-play* data.

^25^https://www.dfl.de/en/news/bundesliga-and-amazon-web-services-to-develop-next-generation-football-viewing-experience/ (accessed September 10, 2020).

## Supplementary Material

We also provide an example of the media live application in German Bundesliga^26^.
